# Quality evaluation of various commercial specifications of Polygoni Multiflori Radix and its dregs by determination of active compounds

**DOI:** 10.1186/1752-153X-6-53

**Published:** 2012-06-08

**Authors:** Zhitao Liang, Ngon-ngon Leung, Hubiao Chen, Zhongzhen Zhao

**Affiliations:** 1 School of Chinese Medicine, Hong Kong Baptist University, Hong Kong Special Administrative Region, People's Republic of China

**Keywords:** Polygoni Multiflori Radix, Decoction pieces, Dreg, Processing, THSG, Emodin, Physcion, HPLC

## Abstract

**Background:**

According to market investigation, two kinds of Polygoni Multiflori Radix decoction pieces with different specifications are commercially available: irregular thick slices (0.7-1.3 cm) and length-wise into thin slices (0.11-0.2 cm). The objective of this study was to evaluate the quality of various samples of Polygoni Multiflori Radix decoction pieces and its dregs.

**Results:**

A simple and reliable high performance liquid chromatographic method was developed for determination the contents of 2,3,5,4′-tetrahydroxystilbene-2-*O*-*β*-*D*- glucopyranoside (THSG), emodin and physcion, which were considered to be potent active ingredients. The results showed that the contents of THSG, emodin and physcion varied in samples of different diameters and thicknesses. The results also indicated the dregs of Polygoni Multiflori Radix still contained a considerable amount of THSG, emodin and physcion.

**Conclusion:**

The various commercial specifications of Polygoni Multiflori Radix sold in the markets did not correlate with their prices, and the dregs of Polygoni Multiflori Radix can be further utilized.

## Background

Polygoni Multiflori Radix is the dried root tuber of *Polygonum multiflorum* Thunb. (Fam. Polygonaceae). Two kinds of decoction pieces of Polygoni Multiflori Radix are recorded in the Chinese Pharmacopoeia: raw and processed [1]. The raw material of Polygoni Multiflori Radix is often processed with black soybean juice or directly steamed until it is brown on all sides; it is then cut into slices, which are sold as “processed” decoction pieces. In the practice of traditional Chinese medicine, raw Polygoni Multiflori Radix is used to counteract toxicity, cure carbuncles, and relax the bowels, while processed decoction pieces are used for replenishing the liver and kidney with vital essence and blood, blackening the hair and strengthening the tendons and bones [1]. 1n recent years, Polygoni Multiflori Radix has been used with satisfactory effects to treat hypertension, hyperlipidemia, coronary heart disease, and alopecia. However, improper usage, excessive dosage or long-term use may result in poisoning. The side effects of liver injury, skin allergy, bleeding in the upper digestive tract, and familial allergy have been reported as the results of misuse [2-5]. Although the mechanisms of such effects have not been clarified, it is important to have a means to evaluate the quality of various specifications of Polygoni Multiflori Radix sold in the markets to provide the useful information for clinical use.

**Figure 1:**
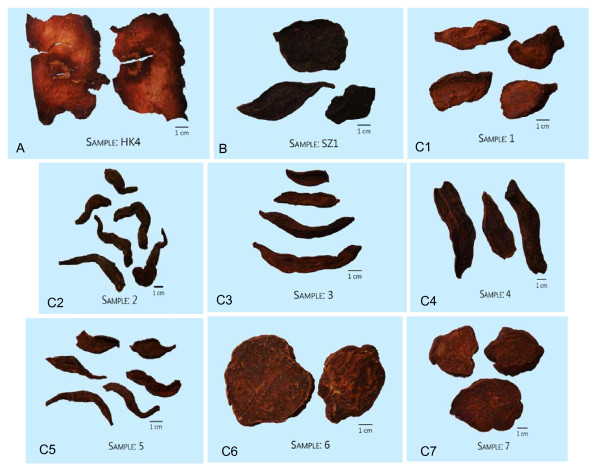
**The various commercial specifications of Polygoni Multiflori Radix decoction pieces in the markets (A: irregular thick slices; B. length-wise into thin slices; C. various commercial specifications in Deqing county, the numbering is consistent to Table**1**).**

Today, most of the Polygoni Multiflori Radix sold for medicinal use is cultivated. Since ancient times, Deqing county, situated in Guangdong province of China, has been famous for the production of high quality with large yield of Polygoni Multiflori Radix. For this reason, the Polygoni Multiflori Radix produced in Deqing county is regarded as superior crude drug. However, from our investigations in local markets and in Deqing county, we found a wide variation in the characteristics of Polygoni Multiflori Radix for sale. Some were cut into irregular thick slices (0.7-1.3 cm) or sections; others were cut length-wise into thin slices (0.11-0.2 cm) (Figure 1). Additionally, the diameter of the Polygoni Multiflori Radix differed, apparently due to whether the cultivation occurred in normal soil or mountain, and cultivation time (Figure 1, Table 1). The prices of Polygoni Multiflori Radix varied according to these differences. Generally, the price of Polygoni Multiflori Radix cultivated in the normal soil for one year was much cheaper than that growing in wild or cultivated in mountain.

**Table 1:** The contents of THSG, emodin and physcion in various specifications of Polygoni Multiflori Radix decoction pieces from Deqing county, Guangdong province, China

**No.**	**Source**	**Contents (mg/g)**			**Price (RMB)**
		**THSG**	**Emodin**	**Physcion**	
C1	Wild	42.6	0.06	0.07	150 Yuan/500 g
C2	Half wild for 5–6 years	68.4	0.10	0.09	120 Yuan/500 g
C3	Cultivated in the mountain for 5–6 years	45.2	0.04	0.06	75 Yuan/500 g
C4	Cultivated in the normal soil for 3–4 years	30.7	0.05	0.06	68 Yuan/500 g
C5	Cultivated in the mountain	60.0	0.26	0.19	55 Yuan/500 g
C6	Cultivated in the normal soil for one year	24.8	0.07	0.08	15 Yuan/500 g
C7	Cultivated in the normal soil for one year	24.3	0.09	0.08	13 Yuan/500 g

**Figure 2:**
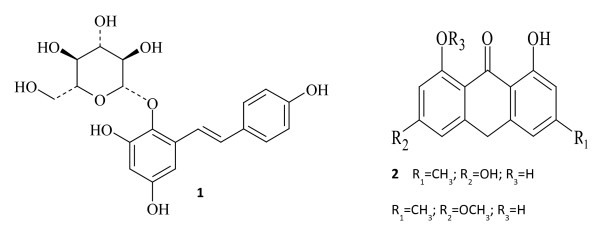
**Chemical structures of active compounds.** 1: 2,3,5,4′-tetrahydroxystilbene-2-O-*β*-D- glucopyranoside; 2: emodin; 3: physcion.

Previous studies have found that Polygoni Multiflori Radix mainly contains anthraquinones, diphenyl ethylene glycosides, amides and chromones, of which 2,3,5,4′-tetrahydroxystilbene-2-*OβD*-glucopyranoside (THSG), emodin and physcion are often used as maker compounds for quality evaluation (Figure 2) [6]. The Chinese Pharmacopoeia specifies that the content of THSG in the raw and processed decoction pieces shall not be less than 1.0% and 0.70%, respectively, while the content of emodin and physcion shall not be less than 0.1% in processed decoction pieces, as determined by high performance liquid chromatography (HPLC). Previous pharmacological studies have also demonstrated that THSG has strong antioxidant activity [7], protective effects on acetic acid–induced colitis and mitomycin C-induced chronic colitis in mice [8] as well as liver injury in peroxidized oil-fed rats [9], anti-atherosclerosis [10] and anti-inflammatory effects [11], etc. Emodin and physcion have been identified as the antioxidant anthraquinones of Polygoni Multiflori Radix [12]. These three compounds can be used as chemical markers for the quality evaluation of commercial Polygoni Multiflori Radix decoction pieces.

In a Chinese clinic, herbs are generally administered as decoctions, prepared from decoction pieces sold in pharmacies. The dregs are the residues of medicinal materials extracted after decoction; these are normally thrown away. Analysis of the active ingredients in the dregs will be helpful to their utilization. In this study, in order to evaluate the commercial Polygoni Multiflori Radix decoction pieces and their dregs, ten batches of Polygoni Multiflori Radix decoction pieces with various specifications as well as their decoction dregs were collected for determination of the contents of THSG, emodin and physcion by HPLC. Although two publications reported the assay of these analytes in Polygoni Multiflori Radix by HPLC [13,14], these two methods were time-consuming and required much solvents. Therefore, a new HPLC method with the advantages of short time and good resolution of analytes was developed to quantify these components in these herbal samples, according to the optimization of sample preparation, mobile phase, wavelength measurement, etc.. The present work is the first report to evaluate various specifications of commercial Polygoni Multiflori Radix decoction pieces. The results are helpful to guide the clinical uses and the public when they buy Polygoni Multiflori Radix in herbal shops or herbal markets according to morphological features.

### Experimental

#### Plant materials

Seventeen samples of commercial decoction pieces of Polygoni Multiflori Radix were collected from Deqing county, Guangdong province of China or purchased from pharmacies in China (Tables 1 &2). All the herbal samples were authenticated by the authors, and the corresponding voucher specimens were deposited in the Bank of China (HK) Chinese Medicines Centre of Hong Kong Baptist University.

**Table 2:** The contents of THSG, emodin and physcion in decoction pieces of Polygoni Multiflori Radix from pharmacies

**No.**	**Source**	**Contents of decoction pieces (mg/g)**			**Contents of dregs (mg/g)**		
		**THSG**	**Emodin**	**Physcion**	**THSG**	**Emodin**	**Physcion**
HK1	Hong Kong; market	0.40	0.05	0.05	-^a^	-	-
HK2	Hong Kong; market	-	-	-	-	-	-
HK3	Hong Kong; market	0.26	-	-	-	-	-
HK4	Hong Kong; market	-	-	-	-	-	-
HK5	Hong Kong; market	0.56	0.09	0.07	-	-	-
HK6	Hong Kong; market	0.25	0.01	0.02	0.11	0.03	0.08
SZ1	Shenzhen city, Guangdong province, China; market	22.6	0.11	0.09	11.2	0.10	0.09
SZ2	Shenzhen city, Guangdong province, China; market	23.2	0.10	0.10	12.0	0.05	0.07
SZ3	Shenzhen city, Guangdong province, China; market	20.5	0.20	0.18	15.8	0.15	0.13
SZ4	Shenzhen city, Guangdong province, China; market	21.7	0.15	0.12	6.0	0.21	0.15

^a^: Not detected.

#### Instrumentation

A CREST 1875HTAG ultrasonic processor (CREST, USA) was used for sample extraction. Binder FDL115 drying oven was used to dry the dregs. A Barnstead Thermolyne Cimarec hotplate was used for heating. HPLC fingerprinting analysis was performed on an Agilent1100 series LC system consisting of a G1311A Quart pump, a G1322A degasser, a G1315A photodiode array detector (DAD) and a G1313A automatic liquid sampler (ALS). Separation was performed at room temperature on an Alltima C_18_ analytical column (250 mm × 4.6 mm, 5 μm, Alltech Associates, Inc. USA) coupled with a C_18_ guard column (7.5 mm × 4.6 mm, 5 μm, Alltech Associates, Inc. USA). The mobile phase system was acetonitrile containing 0.1% formic acid (A)/water containing 0.1% formic acid (B) as well as its gradient elution was 23% A in 0–8 min, 70-75% A in 20–25 min and 100% A at 35 min at a flow rate of 1 ml/min. The injection volume was 20 μl and detection was performed at 290 nm. A microTOF-Q system with electrospray ionization (ESI) source (Bruker Daltonics, Inc. USA) was used for mass spectrometric measurements.

#### Chemicals and reagents

HPLC grade acetonitrile (Labscan, Bangkok, Thailand), analytical grade formic acid (Merck, Germany) and deionized water obtained from a Milli-Q water system (Millipore, Bedford, MA, USA) were used for preparation of the mobile phase. Analytical grade methanol (Labscan, Bangkok, Thailand) was used for preparation of standards and sample extraction. Analytical grade chloroform (Labscan, Bangkok, Thailand) was also used. Reference compounds of 2,3,5,4′-tetrahydroxystilbene-2-O-β-D- glucopyranoside (1), emodin (2) and physcion (3) (all with purities > 97%) were purchased from the National Institute for the Control of Pharmaceutical and Biological Products, China (Batch No. 110844–200505, 110756–200110 and 110758–200610, respectively).

#### Preparation of standard and sample solutions

The three reference compounds (1–3) were accurately weighed. THSG and emodin were dissolved in methanol while physcion was dissolved in chloroform to produce standard stock solutions. The stock solution was diluted to yield a series of standard solution in the concentration range of 16–1600 μg/ml, 0.2-40 μg/ml, and 0.2-48 μg/ml for THSG, emodin and physcion, respectively. Samples of herbal materials were ground into fine powder then passed through a 20 mesh (0.9 mm) sieve. Sample powder (0.3 g) was accurately weighed and transferred into a 100 ml round bottom flask. Methanol (50 ml) was added and refluxed for 120 min. When cool, the methanol was added to compensate for weight loss. After filtering through a 0.45 μm filter membrane, the filtrate was ready to be used as a test sample. Sample and sample duplicate were simultaneously prepared and analyzed. The average value of analytes in the sample and sample duplicate was calculated as their amounts in a specific solid sample.

The decoction pieces of processed Polygoni Multiflori Radix collected from pharmacies were immersed in water for 30 min and boiled for 1 hour according to the typical procedure for decocting herbal samples as recommended by Chinese medicine clinics. After decoction, the decoction pieces of Polygoni Multiflori Radix as dregs were dried in the oven; sample solutions were then prepared following the above steps.

## Results and discussion

### Evaluation of extraction method

The choices of extraction solvents, time and methods were optimized. The same herbal material was extracted by reflux for 60 min using 50 ml of various solvents in various concentrations: water (then extracted by acetate ethyl), 30% acetone water solution, 40%, 60%, 80% methanol and total methanol. The results showed that methanol extraction produced the best yield for THSG, emodin and physcion. The extraction conditions were further optimized in terms of extraction time at 60, 120 and 180 min with methanol. The results indicated there was no significant difference of the contents of THSG, emodin and physcion occurred between 120 min and 180 min extraction time. Considering that the prolonged extraction time may bear the risk of sample deterioration, 120 min extraction time was chosen. Additionally, the extraction methods of sonication and Soxhlet by methanol were compared. Using the reflux extraction method, the contents of THSG, emodin and physcion were 30.8 mg/g, 0.12 mg/g and 0.17 mg/g, respectively. For Soxhlet extraction method, the contents of THSG, emodin and physcion were 25.0 mg/g, 0.12 mg/g and 0.15 mg/g, respectively while 15.4 mg/g, 0.10 mg/g and 0.13 mg/g for sonication method, respectively. Overall it could be concluded that the extraction scheme as outlined above was optimized for the analysis.

### Method validation

Method validation parameters included linearity, recovery, repeatability, precision, stability and quantitation limit. Because the peak intensities of THSG, emodin and physcion were remarkably different among the collected samples, peaks of THSG, emodin and physcion were identified in the chromatograms of the tested samples by comparing them with reference standards with respect to retention time, on-line UV spectra and LC-MS data. The protonated ions of THSG (407.1 *m/z*, [M + H]^+^), emodin (271.1 *m/z*, [M + H]^+^) and physcion (285.1 *m/z*, [M + H]^+^) were obtained in standard and sample solutions.

Satisfactory linearity for the analysis of each compound was obtained. Linearity was examined with selected concentration range with 6 levels. The calibration curves were constructed by plotting the peak areas (mAU) of the analytes versus the concentration (μg/ml). The linear regression equation and correlation coefficient (R^2^) were *y* = 10.127*x* + 83.395 (R^2^ = 0.9998, n = 6) for THSG, *y* = 64.009*x* + 2.8734 (R^2^ = 0.9999, n = 6) for emodin and *y* = 56.913*x*-8.854 (R^2^ = 0.9998, n = 6) for physcion. These equations were then applied to calculate the amount of these analytes in sample extracts.

Recovery study was conducted on a sample spiked with about 100% of known amounts of THSG, emodin and physcion in the samples with 3 replicated analyses. The spiked samples were extracted and the amounts of these analytes were quantified. All recoveries were in the range of 99.8-105.8%. The results showed that the average recoveries were estimated to be 105.0 ± 0.7% (mean ± SD, n = 3) for THSG, 100.8 ± 0.8% (mean ± SD, n = 3) for emodin and 100.40 ± 0.6% (mean ± SD, n = 3) for physcion.

Method repeatability was evaluated by five replicated analyses of herbal samples. The RSDs of the content of THSG, emodin and physcion in five replicated herbal samples was 0.80%, 0.97% and 0.01%, respectively.

Method precision was investigated by repeatedly analyzing the same set of sample solution, the values of relative standard deviations (RSDs) were 0.40%, 0.27% and 0.20% (n = 5) for THSG, emodin and physcion, respectively.

Stability testing was performed on a sample solution after standing for 0, 2, 4, 8, 12, 24, 48 and 72 h. The results showed that the RSD of THSG, emodin and physcion was 1.39%, 1.31% and 1.30% (n = 8), respectively.

The quantitation limit evaluated by a signal-to-noise ratio of about 10:1 for the sample solution, was determined to be 0.58 μg/ml, 0.09 μg/ml and 0.15 μg/ml for THSG, emodin and physcion, respectively.

### Comparison of the contents of THSG, emodin and physcion in various raw Polygoni Multiflori Radix decoction pieces

**Figure 3:**
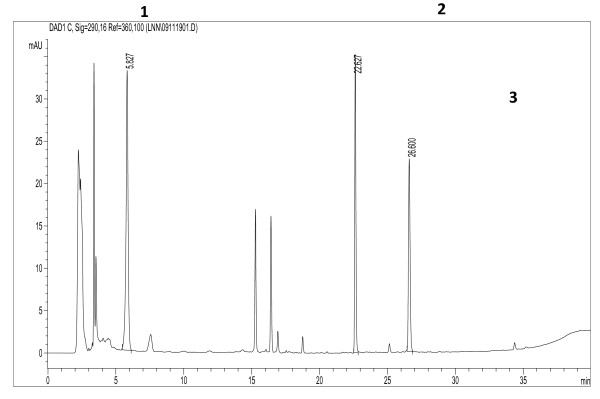
**Typical HPLC chromatogram of Polygoni Multiflori Radix decoction pieces from Deqing county, Guangdong province, China**. Peak1: 2,3,5,4′-tetrahydroxystilbene-2-O-*β*-D- glucopyranoside; Peak 2: emodin; Peak 3: physcion.

In the chromatograms, the chromatographic peaks of THSG, emodin and physcion were well separated (Figure 3). Compared with the reference compounds, the on-line UV spectra and MS data, chromatographic peaks 1, 2 and 3 were unambiguously identified, respectively, as THSG, emodin and physcion.

Deqing county is considered to be one of the production areas for genuine Polygoni Multiflori Radix. In the local herbal markets, several grades of commercial decoction pieces of raw Polygoni Multiflori Radix were sold at different prices (Table 1). Different grades have different quality; generally, higher grade means higher quality. The results showed that the content of THSG in ascending order was C7 < C6 < C4 < C1 < C3 < C5 < C2. The total contents of emodin and physcion were C3 < C4 < C1 < C6 < C7 < C2 < C5, but there were no big differences between samples C1 (0.13 mg/g), C3 (0.10 mg/g) and C4 (0.11 mg/g); furthermore, the contents of samples C2 (0.19 mg/g), C6 (0.16 mg/g), and C7 (0.17 mg/g) were also very close. Among the collected samples, the samples cultivated in the mountain had higher contents of THSG, emodin and physcion than those cultivated in the normal soil. According to the prices for the various commercial specifications of Polygoni Multiflori Radix sold in markets, it was clear that the contents of THSG, emodin and physcion did not always correlate with price. For example, sample 5 contained higher contents of THSG, emodin and physcion but it was cheaper than samples 1, 3 and 4. Distinctly, the classification of various grades of Polygoni Multiflori Radix actually did not distinguish relative quality.

As well know, the quality of herbal medicines can be affected by many factors, such as geography, seasonal variation, cultivation, harvesting, storage and post-harvest treatment. But the distribution of herbal metabolites in herbal tissues was consistent. Our previous studies have found that the THSG and anthraquinones mainly distributed in the cork and cortex [15]. Thus the decoction pieces of raw Polygoni Multiflori Radix with more area of cork and cortex have better pharmaceutical quality and the current finding validated the conclusion.

### Comparison of the contents of THSG, emodin and physcion in processed Polygoni Multiflori Radix decoction pieces collected from pharmacies before and after decoction

In order to clarify the situation of Polygoni Multiflori Radix decoction pieces sold in pharmacies, ten batches of samples were collected for analysis. The results showed that the contents of THSG, emodin and physcion in the thin slices were low, and sometimes undetectable (Table 2). In contrast, those irregular thick slice samples contained higher contents of THSG, emodin and physcion. After decoction, the content of THSG decreased while the contents of emodin and physcion varied. In some samples, the contents of emodin and physcion decreased after decoction but in others they increased. However, distinctly, the dregs of Polygoni Multiflori Radix still contained a big amount of THSG, emodin and physcion. Therefore, the irregular thick slice samples of Polygoni Multiflori Radix used in clinic should be boiled longer than an hour. Also, the dregs could be boiled again for treatment or further utilized, such as extracting the residual active ingredients for second development.

It have been demonstrated that the chemical constituents of crude herbal medicines can be changed dramatically during processing [16]. Previous investigations have demonstrated that the contents of emodin and physcion in processed Polygoni Multiflori Radix were found to be higher than those in raw materials because the anthraquinones glycosides transferred into free anthraquinones. But the contents of anthraquinones glycosides and stilbene glucoside in raw Polygoni Multiflori Radix were significantly higher than those in the processed samples [17,18]. The present finding indicated that the degree of processing in those thin and irregular thick slice samples was different and the decocting influenced the ratios of free anthraquinones and anthraquinones glycosides. Moreover, the contents of emodin and physcion in most of processed decoction pieces did not reach the requirements specified in the Chinese Pharmacopoeia. Therefore, the manufacturing practice for processing Polygoni Multiflori Radix should be improved, and more attention should be paid to quality assessment of decoction pieces sold in the herbal markets and pharmacies

## Conclusion

A convenient method has been developed for the quantitative analysis of the contents of THSG, emodin and physcion in Polygoni Multiflori Radix. The various commercial specification of Polygoni Multiflori Radix did not correctly distinguish their quality and prices. The decoction dregs still contained a big amount of THSG, emodin and physcion and should be further utilized.

## Abbreviations

THSG, 2,3,5,4′-tetrahydroxystilbene-2-O-β-D- glucopyranoside; HPLC, High performance liquid chromatography; DAD, Photodiode array detector; ALS, Automatic liquid sampler; ESI, Electrospray ionization.

## Competing interests

The authors declare that they have no competing interests.

## Authors’ contributions

ZZZ initiated and all authors designed the study. The sample extraction was conduct by NNL. The method developments were conducted by ZTL who drafted the manuscript. All authors contributed to the data analyses and to finalizing the manuscript. All authors have read and approved the final version.
